# 3D Printing Approach to Valorization of Agri-Food Processing Waste Streams

**DOI:** 10.3390/foods12010212

**Published:** 2023-01-03

**Authors:** Kandasamy Suppiramaniam Yoha, Jeyan Arthur Moses

**Affiliations:** Computational Modeling and Nanoscale Processing Unit, National Institute of Food Technology, Entrepreneurship and Management—Thanjavur, Ministry of Food Processing Industries, Government of India, Thanjavur 613005, Tamil Nadu, India

**Keywords:** food-processing waste, 3D printing, sustainable food processing, waste-to-wealth, value addition, circular economy

## Abstract

With increasing evidence of their relevance to resource recovery, waste utilization, zero waste, a circular economy, and sustainability, food-processing waste streams are being viewed as an aspect of both research and commercial interest. Accordingly, different approaches have evolved for their management and utilization. With excellent levels of customization, three-dimensional (3D) printing has found numerous applications in various sectors. The focus of this review article is to explain the state of the art, innovative interventions, and promising features of 3D printing technology for the valorization of agri-food processing waste streams. Based on recent works, this article covers two aspects: the conversion of processing waste streams into edible novel foods or inedible biodegradable materials for food packing and allied applications. However, this application domain cannot be limited to only what is already established, as there are ample prospects for several other application fields intertwining 3D food printing and waste processing. In addition, this article presents the key merits of the technology and emphasizes research needs and directions for future work on this disruptive technology, specific to food-printing applications.

## 1. Introduction

Food loss and food waste are aspects of global concern. Specifically, agri-food processing waste streams pose a serious challenge to the environment. Industrial waste streams can be either solid–liquid fractions or their blends. The impact of poor disposal and handling practices is increasingly being understood, and recent years have witnessed a surge in policies specific to such issues. Judicious usage of such processing waste streams is linked to sustainable food processing. For example, agri-food processing wastes consist of nutrients and bioactive compounds; reintroducing them into the value chain is a strategy consistent with the zero-waste circular economy [1]. In addition, the waste or by-products of one food-processing sector can be used as a resource for another. Hence, a biorefinery approach can provide a way to encourage an emerging technological revolution with a sustainability focus. Importantly, the conversion of waste biomass to various compounds facilitates better waste management.

According to the recently issued 2022 global report on food crises, there were 135 million individuals who experienced acute food insecurity in 2019, which increased to 193 million in 2021. This makes food waste and loss a moral issue that will not be considered acceptable [2]. The statistics presented above are quite concerning, and therefore need to focus on value-added food that is easy to produce through waste valorization and meets all dietary requirements. In addition, food loss/waste contributes 8–10% of the world’s greenhouse gas emissions [3]. Hence, concerns about the socioeconomic and environmental consequences of food waste have focused more attention on the issue.

For sustainable development, the reduction of food loss/waste or reutilization of waste (valorization) is necessary to end world hunger and provide food security and adequate nutrition for everyone [4]. The Food and Agriculture Organization (FAO) takes the lead in this area and supports the coordinated effort required to effect it. Though preventing food waste is preferred, a significant amount of food waste is unavoidable as the world’s economy grows. Agri-food processing wastes consist of nutrients and bioactive compounds and reintroducing them into the value chain is a strategy that is consistent with the zero-waste circular economy [1]. The waste or by-products of one food processing sector can be used as a resource for another. Hence, biorefineries can provide a way to encourage an emerging technological revolution with a sustainability focus. The conversion of waste biomass to various compounds aids in the reduction of waste to be disposed of.

Among various trends in the food industry, 3D printing is emerging as a novel approach, capable of changing the way food is manufactured. Particularly, in the past decade, an array of applications has been established, ranging from their scope in developing meals tailored to personalized nutrition to the delivery of nutraceutical ingredients. The technology supports food model prototyping, is highly versatile, supports digitization, and has the possibility of simplifying manufacturing processes and supply chains [5].

**Figure 1:**
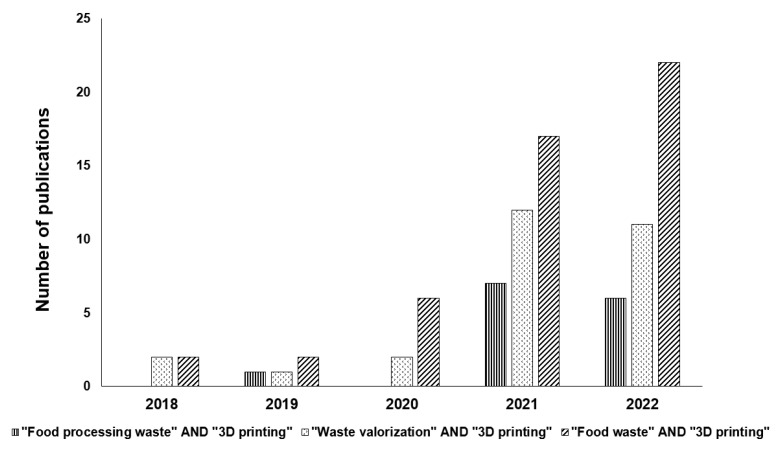
Publication trends in the application of 3D printing using food waste. Source: Online Scopus database (www.scopus.com) using the keywords (“Food processing waste” AND “3D printing”; “Waste valorization” AND “3D printing”; “Food waste” AND “3D printing” (accessed on 22 December 2022).

As an additive manufacturing process, 3D printing can support the concept of zero waste in the food industry and can also be used for better utilization of processing waste streams. Figure 1 illustrates how 3D printing is becoming more prevalent from the standpoint of valuing food waste. It indicates the emergence of the application of 3D printing technology for utilizing food waste.

For example, waste or by-products from the food processing industry (such as milling fractions, fruit and vegetable peels, and other waste products) can be converted into a dried powder form that can be added to the printing material supply [6,7]. Accordingly, the focus of this article is to summarize recent works demonstrating the scope of applying 3D food-printing technology for the valorization of agri-food processing waste streams and presenting emerging avenues for research and development.

## 2. Agri-Food Processing Wastes in the Food Supply Chain

The most common types of food-processing waste are offcuts, trimming waste, defective products, and by-products from the food manufacturing process. Food manufacturers and processors need to use resources as efficiently as possible because there is a high demand for a wide range of products [8]. Hence, unused/leftover edible parts from processing may be diverted for other food or high-value applications. The concept of reuse can minimize the amount of food waste being discarded. From an economic point of view, distributing excess materials, wastes, and by-products to other manufacturing industries as raw materials for food production will prove beneficial. 

Food processing waste streams can contain edible fractions that can be directly processed and reused in food product development, subject to testing as safe in physical, chemical, and microbiological aspects. An interesting example is a scope of using grape pomace as a raw material for the manufacture of baked foods [9]. Waste streams can also contain certain high-value ingredients that can be extracted, isolated, and purified for specific applications; for example, nutraceutical ingredients from fish processing waste [10]. Several works have demonstrated that food wastes can be used as raw material for the development of health supplements, antimicrobials, therapeutic ingredients, bioactive enzymes, polysaccharides, hydrocolloids, peptides, single-cell proteins, pigments, and aromatic compounds [11]. Table 1 represents various approaches to the valorization of agri-food waste for the development of valuable products/high-value resources. Wastes can also have inedible fractions that can be used for the development of biopolymers [12,13]. Recently, anacardic acid from cashew-processing waste was explored for active packaging applications [14]. In addition, instead of merely dumping wastes in landfills, several reports demonstrate their possible use in energy production [15]. Overall, there is tremendous scope for their recovery and usage in a range of applications.

## 3. 3D Printing Approach to Waste Valorization

According to the statistical report “Global 3D Food Printing Market: Focus on Technology (Fused Deposition, Selective Sintering, and Powder Bed Binder Jetting), Vertical (Commercial, Government, and Hospital), and Food Type (Confections, Meat, and Dairy)—Analysis and Forecast 2018–2023”, the 3D food printing market is projected to reach USD 525.6 million by 2023. In general, however, prospects for waste valorization remain underexplored as of today. One interesting application is the scope for using processed fractions of food processing waste streams in blends with regular printing material. In particular, this would be of benefit in cases wherein their addition can improve the rheological behavior of the material supply, thereby making it suitable for extrusion-based 3D food printing. At the same time, however, their addition may negatively affect printability. This is because some food waste fractions, for example, those rich in fiber, can be challenging to 3D print [36]. However, if successfully optimized, they can prove extremely beneficial. One indicative example is the scope of such materials being rich in prebiotic ingredients. Dietary fiber is known to enhance the nutrient absorption ability of the gut [37]. Its incorporation into the printing material supply, particularly types loaded with probiotics, can offer symbiotic effects that are extremely beneficial for the gut and other health benefits [38]. In addition to their fiber content, fruit and vegetable waste streams, including peel, seed, skin, rind, and pomace, are rich sources of bioactive compounds such as vitamins, proteins, phenolics, carotenoids, and enzymes [39]. Unlike most conventional food manufacturing approaches, 3D printing can provide high levels of customization and exceptionally high aesthetic appeal. This can be exploited for the development of nutrient-dense snacks and foods from ingredients (including food waste fractions) that are often otherwise not viewed as desirable. 

3D-printed foods can also be designed to deliver essential micro- and macronutrients. The United States military has already announced intentions to use 3D food printing for simple food preparation on the front lines and personalized nutrient intake for soldiers [40]. In this regard, according to the statistical report “Functional Foods Market Size, Share & Trends Analysis Report By Ingredient (Carotenoids, Prebiotics & Probiotics, Fatty Acids, Dietary Fibers), By Product, By Application, and Segment Forecasts, 2019–2025”, the global functional foods market size is projected to reach USD 275.77 billion by 2025. It is anticipated to expand at a compound annual growth rate (CAGR) of 7.9% during the forecast period. Increasing demand for nutritional and fortifying food additives is one of the major growth drivers. The increasing trend of consuming these products is expected to maintain throughout the forecast period, thereby favoring market growth [41].

It is also possible to optimize the design and manufacturing practices of 3D food printing for high-volume production processes. Given its high flexibility and subtlety, it is easy to fabricate a customizable design with intrinsic complex structures using a computer-aided design (CAD) software programming, and it can be quickly scaled up to meet the demand for desired products [42]. Different technologies such as extrusion printing, binder jet printing, stereolithography, selective sintering, fused deposition modeling, and inkjet printing have been developed for 3D food structure development [43,44]. Based on the type of feed material (liquid/paste/solid), specific techniques can be adopted for 3D food design. Among the different techniques, extrusion printing is the most widely explored type of food 3D printing.

## 4. Important Parameters of the 3D Printing Process

### 4.1. Material Parameters

The printability of a material is determined by feed material properties such as type of material, particle size, diffusion/dispersion pattern, binding properties, surface tension, and extrusion behavior. The rheology and texture of the material are the most crucial parameters in the context of extrusion 3D printing and explain the material’s behavior and properties. 

The rheology of feed material (solidification or gelation upon cooling) defines the binding of the deposited layer. Shear stress, shear rate, and apparent viscosity define the rheology of a printing material [45]. The pasting profile is expressed in terms of peak viscosity, pasting temperature, holding strength, breakdown viscosity, final viscosity, and setback viscosity. The viscosity of the food feed material is a critical parameter for extrusion through a fine nozzle. Given this, the rheology of the food feed material can be altered using different additives [46]. For instance, 3D printing of egg white and yolk was achieved with rice flour as an additive to obtain the required rheological properties [47]. The rheological properties of such non-natively printable materials can also be altered by adding fractions from food waste streams.

The strength of a material can be studied by performing a texture analysis. Solid and soft food materials like dough, processed cheese, etc., can be printed using soft material extrusion [48]. The melting extrusion technique is adopted for food materials such as chocolate, where the working temperature is generally 5 to 10 ℃ above the feed material melting point. Upon cooling, crystallization of the feed occurs and it is deposited as a solid 3D structure [49]. For hydrogel-forming feed materials such as polysaccharides, proteins, and polymers with high water-holding capacity, simple extrusion can be easily adapted to create 3D structures. The gel-forming mechanism and rheological properties of polymer feed are critical to controlling the gelation and formation of self-supporting gels before the deposition of a consecutive layer. Different gel-forming mechanisms such as chemical cross-linking, complex coacervation, and ionotropic cross-linking are used in hydrogel feed material preparation for 3D printing [50]. 

### 4.2. Process/Printing Parameters

Printability often becomes challenging to define, as it is a complex term encompassing the effects of nozzle diameter, nozzle tip-to-target distance (nozzle height), printing speed, extrusion rate, etc. [51]. In short, nozzle diameter or size determines the thickness of the printed line and layer height of 3D-printed objects, and the distance between the nozzle tip-to-target plate, known as nozzle height, must be optimized since it has a direct impact on the structure of 3D-printed objects. The feed material can be printed with the desired shape and structure by assessing the appropriate/optimized extrusion rates; an increased extrusion rate can provoke the spreading or smudging of the material. Optimization of printing speed is necessary for relation to other printing parameters and is defined by the speed of the extrusion motor; an increased printing speed results in fragmented lines, while inadequate printing speed leads to wavy strand formation, resulting in over-deposition of printing material. On the whole, optimization of 3D printing conditions may become challenging, and there is scope for modeling-based and machine-learning approaches to minimize the number of trial-and-error runs in identifying optimal conditions. 

### 4.3. Post-Processing Characteristics

3D-printed products, particularly those that are cold-extruded, can benefit from post-processing. These benefits can be in terms of improvements in structural stability, shelf life, palatability, and/or digestibility. However, inappropriate post-processing types and conditions can adversely affect the quality of 3D-printed structures [38,52]. As the post-processing operation decides the quality and acceptability of the final product, it is crucial to optimize the ideal post-processing method and its operational parameters for specific products in order to retain the desired visual, mechanical, sensorial, and nutritional attributes. Different post-processing operations such as baking, steaming, drying, frying, and other conventional food-processing approaches can be employed for edible 3D-printed products. In contrast, drying, priming, coating, and curing process using plasma/laser/ultraviolet rays and gelation by enzymatic/thermal/chemical cross-linking, etc., are common post-processing operations for inedible 3D-printed objects [53,54,55].

## 5. Application Range

### 5.1. Development of 3D-Printed Foods from Waste Fractions

3D printing is perceived as a sustainable technology that can transform low-value agri-food processing waste into high-value 3D-printed functional foods [56]. The waste produced from the primary food processing line, such as peels, shreds, stalks, fines, and pomace from the vegetable and fruit processing sector; offcuts, trimmings, shell, skin, and bone wastes from the meat and fish processing sector; and broken grains, bran, husks, and other fractions from the mill processing sector can potentially be utilized as direct resources or additive ingredients for 3D food printing [57]. However, it is crucial to take into account any potential risks associated with the utilization of agri-food processing wastes. Hence, it is necessary to standardize the selection of food waste to avoid the risk of microbial contamination or toxic substances from food waste. 

Some research studies that have extensively explored the potential of using 3D printing technology for waste-to-wealth conversion are summarized in this section.

#### 5.1.1. Incorporation of Potato-Processing By-Products into 3D-Printed Yam Snacks

**Figure 2:**
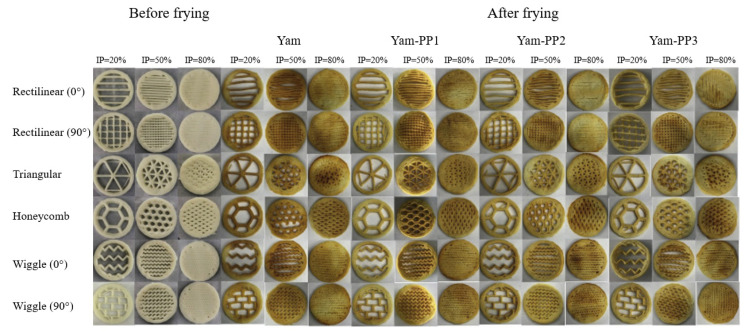
Effect of the addition of potato-processing by-product (PP) on 3D-printed yam snacks before and after post-processing in various combinations (yam/PP = 9:1, 8:2, and 7:3) with different infill percentage levels (20%, 50%, and 80%) “Reprinted/adapted with permission from Ref. [58]. Copyright © 2020, Elsevier”.

The by-products from the potato industry are rich in fiber and they can be used as an additive in other food formulations to improve their mechanical stability through texture modification. Feng et al. [58] developed a 3D-printed yam snack with the incorporation of by-products obtained from potato processing. Furthermore, they explored the printability and post-processing (air-frying) stability of fiber-enriched 3D-printed yam snacks. The visual appearance of each 3D-printed yam snack before and after post-processing is depicted in Figure 2. The results showed that the mixture of yam powder and potato-processing by-product at a mass ratio of 7:3 had a better mechanical strength.

The addition of high fiber into the formulation resulted in increased dietary fiber and reduced extrusion swelling in the 3D-printed product. The infill structures and levels of printing had an impact on the weight and porosity of the printed products. Low infill levels developed products with high porosity and lightweight. Therefore, the hardness of post-processed 3D-printed products decreases as porosity increases. In addition, Feng et al. reported that the strong barrier film formed by the high-fiber potato by-product supported the stability of the product during post-processing. Recovering these types of food processing wastes by producing innovative foods results in a green and sustainable valorization process. Overall, 3D printing is a promising eco-friendly technology for creating innovative textures with unique flavors.

#### 5.1.2. 3D Printing of Fruit Wastes Incorporated Foods

**Figure 3:**
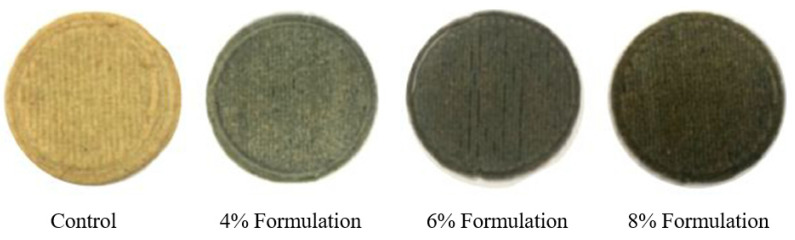
3D-printed functional cookies with different levels of grape pomace powder “Reprinted/adapted from Ref. [6]. Creative Commons CC-BY license, Copyright © 1969, Elsevier”.

Grape pomace contains a variety of nutrients, such as natural antioxidants, phytonutrients, and dietary fibers, which can be used to enhance the modern diet. By utilizing grape pomace and broken wheat, Jagadiswaran et al. [6] developed functional cookies using 3D printing. Broken wheat from milling industries is high in protein, fiber, and other micronutrients. The flour made from broken wheat can be an effective substitute for flour in typical cookies. A crucial component of cookie production is dough rheology, which regulates the mechanical characteristics and flow behavior of the dough. Effective 3D printing requires material supply optimization through rheology studies. Jagadiswaran et al. [6] studied the compatibility of a material supply made up of grape pomace and broken wheat flour and explained the rheological characteristics of the dough. In addition, they optimized the printing process of the dough, made up of a mixture of broken wheat flour with various concentrations (0, 4, 6, and 8%) of grape pomace powder (Figure 3). The optimal printability was achieved by printing with a 1.28 mm nozzle diameter at 600 rpm extrusion motor speed and 400 mm/min printing speed. Textural characteristics and sensory attributes were investigated after post-processing. The results revealed that the functional cookies with 6% grape pomace that underwent post-processing by baking for 12 min at 130 ℃ were more palatable, with enhanced shape stability. Similarly, Leo et al. [59] developed vitamin and nutrient-rich 3D-printed snacks with the incorporation of orange peel waste. These waste valorization studies provided insightful information for subsequent research on waste conversion into value-added products with high consumer acceptance using 3D printing.

#### 5.1.3. 3D Printing of Vegetable Wastes Incorporated Foods

**Figure 4:**
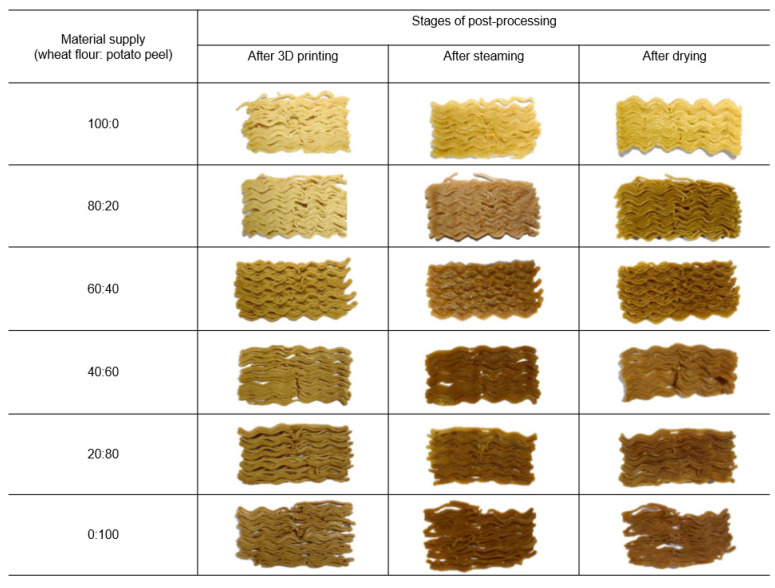
Optimization of material supply for 3D-printed noodles from potato peel waste “Reprinted/adapted with permission from Ref. [7]. Copyright © 2021, exclusive license to Springer Science Business Media, LLC, part of Springer Nature”.

Potato peel has been identified as a significant source of dietary fiber, accounting for 40–45% of its dry weight. It is also a good source of starch, protein, and phenolic compounds. It is regarded as a zero-value waste. Muthurajan et al. [7] developed ready-to-cook 3D-printed noodles from potato peel waste and wheat flour. They have reported that the printability of potato peel fine fractions with particles smaller than 0.125 mm was superior compared to coarse fractions with particle size > 0.125 mm due to less fiber content (1.2%) in fine fractions. Enhanced printability of the material supply was achieved at the ratio of 60:40 (wheat flour:potato peel powder) using a nozzle size of 1.28 mm at 6 bar pressure with a printing speed of 600 mm/min and extrusion motor speed of 600 rpm (Figure 4). Post-processing of the printed noodles was optimized with subsequent steaming (5 min) and drying (68 ℃ for 2.5 h) processes. The calorific value of the prepared noodles was reported as 414.39 kcal per 100 g. Muthurajan et al. also compared the cooking quality, texture, and consumer acceptance with commercial noodles. Another recent study utilized the wastes from green leafy vegetables such as kale stalks and spinach stems for the development of 3D-printed soft and easy-to-chew foods for dysphagia (swallowing difficulty) patients [60]. These studies provide a novel perspective on how to better utilize food waste streams.

#### 5.1.4. 3D-Printed Salmon Gelatin Gel from Valorized Salmon Skin Waste

**Figure 5:**
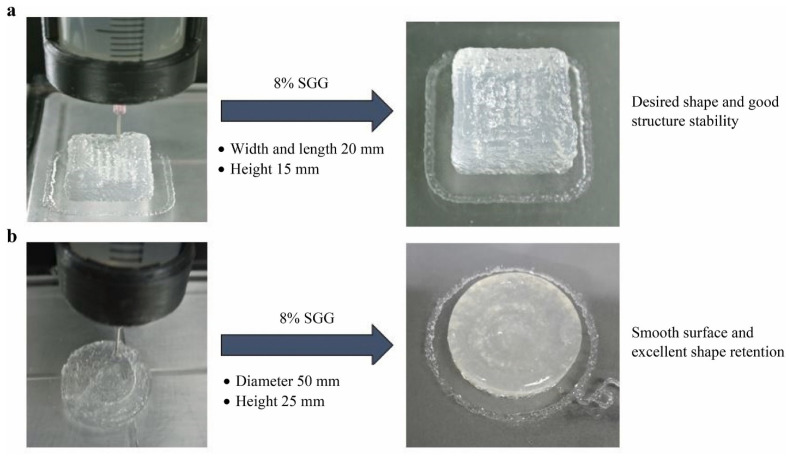
3D-printed salmon gelatin gel (SGG) with the desired shape. (**a**) cube, (**b**) cylinder. “Reprinted/adapted with permission from Ref. [61]. Copyright © 2021, Elsevier”.

Gelatin has typically been extracted from porcine and bovine skin, but interest in marine gelatin is steadily growing. Carvajal-Mena et al. [61] effectively utilized the gelatin gel from salmon skin for the development of 3D-printed salmon gelatin cubes. The printability of the salmon gelatin gel (SGG) at five different concentrations (2, 5, 8, 11, and 14%) was optimized by examining its rheological characteristics, gelling properties, extrudability, and dimensional stability. The results showed that enhanced printability was achieved at 8% SGG concentration using a nozzle size of 0.7 mm with an extrusion speed of 20 mm/s, at a fixed initial layer height and height between layers of 0.5 mm and 0.65 mm, respectively (Figure 5). The temperature conditions were optimized for better stability and the printing and printing bed temperatures were set at 15 ℃ and 6 ℃, respectively. This research provides important information on the suitability and printability of SGG for 3D food-printing applications.

#### 5.1.5. 3D Printing of Foods Incorporating Soybean By-Product (Okara) 

**Figure 6:**
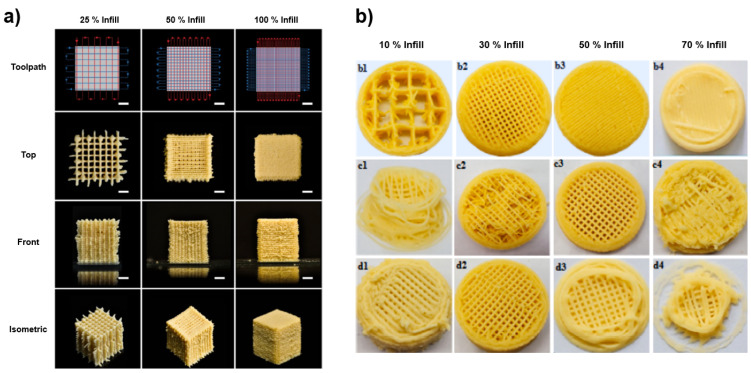
3D-printed (**a**) okara cubes at different infill levels “Reprinted/adapted with permission from Ref. [62]. Copyright © 2021, American Chemical Society” and (**b**) wheat-flour cookies incorporating okara fiber at different infill levels (b1 = 10%, b2 = 30%, b3 = 50%, b4 = 70%), nozzle diameters (c1 = 0.4 mm, c2 = 0.6 mm, c3 = 0.8 mm, c4 = 1 mm) and printing speeds (d1 = 25 mm/s, d2 = 50 mm/s, d3 = 75 mm/s, d4 = 100 mm/s) “Reprinted/adapted with permission from Ref. [63]. Copyright © 2021, Elsevier”.

Okara is the major by-product of soy product manufacturing industries. Lee et al. [62] developed 3D-printed okara snacks without rheology modifiers. They studied the printability of okara formulations at various concentrations (25, 33, and 50% *w*/*w*) of fine okara powder (<100 μm) with distilled water and observed the layer consistency at different infill percentages (25, 50, and 100%) (Figure 6a). The formulation at 33% concentration of okara powder showed better rheological properties—yield stress and storage modulus of 200 ± 40 and 23,300 ± 300 Pa, respectively—and observed increased hardness (47.00 ± 4.58 g) with increased infill percentage (100%). In another study, insoluble dietary fiber was extracted from okara using different treatments (ultrasound and high-speed homogenization) and incorporated into wheat flour cookies at different concentrations (2, 4, 6, and 8%) to increase the dietary fiber content [63]. Better printability was obtained from the addition of 6% okara fiber with 30% infill rate, 0.8 mm nozzle diameter, and 50 mm/s printing speed (Figure 6b). These studies demonstrated the feasibility and printability of okara for the development of fiber-rich 3D-printed snacks.

#### 5.1.6. 3D-Printed Surimi from Cod By-Products

**Figure 7:**
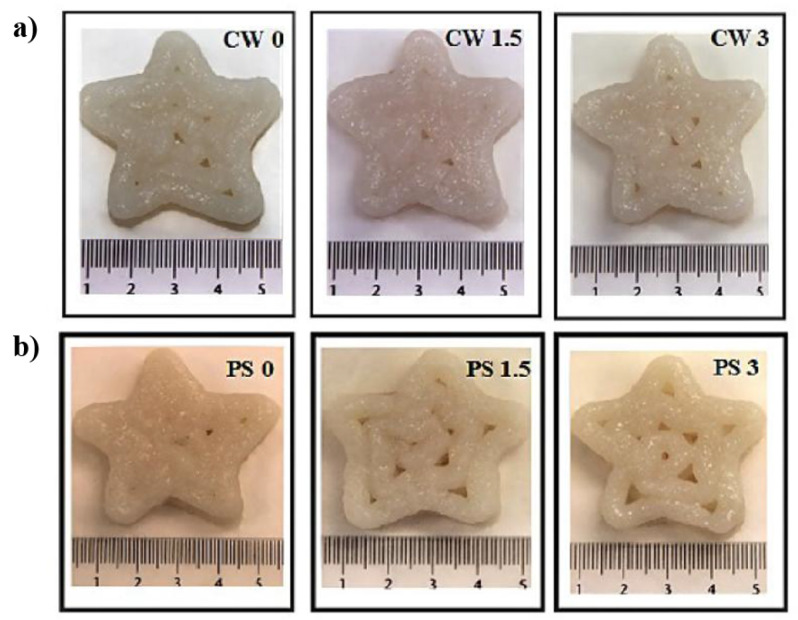
3D-printed surimi from cod by-products (**a**) conventionally washed and (**b**) pH-shift processed with the addition of 0, 1.5, and 3.0% salt “Reprinted/adapted with permission from Ref. [64]. Copyright © 2019, John Wiley & Sons”.

Gudjónsdóttir et al. [64] developed protein-rich 3D-printed surimi from cod (*Gardus morhua*) by-products. They prepared the surimi paste using different methods—conventional washing and a pH-shift process and then stored it at −25 °C (0, 4, and 7 days). They studied printability using a 4 mm diameter nozzle with the addition of 0, 1.5, and 3.0% salt (Figure 7). The 3D-printed samples were subjected to steam cooking at 90 ℃ for 20 min and refrigerated overnight for an optimal setting. The printability and characteristics of the 3D-printed surimi were investigated using low-field nuclear magnetic resonance (LF-NMR) and chemometrics, respectively. They also analyzed the effect of the pH-shift process, cold storage, and salt treatment. The results reported that the fresh sample had better characteristics compared to other counterparts. Increased salt concentration showed significant myofibrillar swelling and a gelling effect in conventionally washed and pH-shift processed surimi, respectively.

### 5.2. Utilizing Inedible Food Waste Fractions

Several food wastes are rich in inedible fractions. While one promising application is the scope of developing biopolymers, recent works have demonstrated the capability of 3D printing technology to fabricate customized food packaging. This section summarizes these works and highlights the scope for the development of replacements for single-use plastics and the design and development of biodegradable cutlery (and possibly edible cutlery and packaging).

#### 5.2.1. 3D-Printed Food Packaging from Rice-Husk Fractions

**Figure 8:**
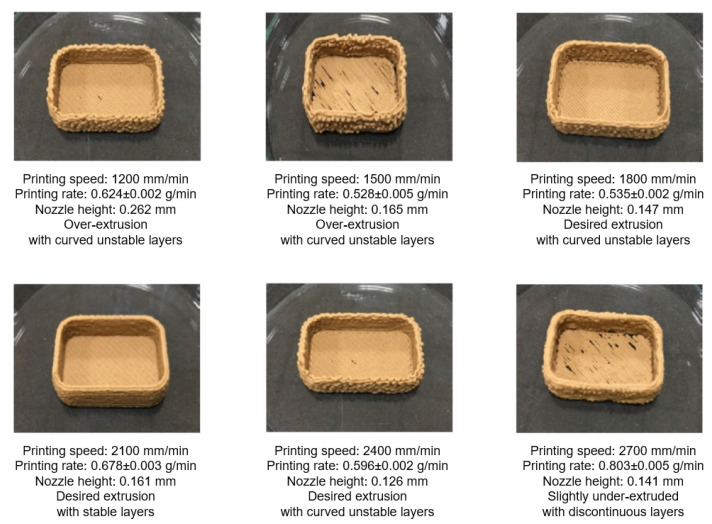
Optimization of material supply for 3D-printed food casings from milled rice husk with 1% guar gum using a nozzle diameter of 0.82 mm at 300 rpm motor speed and 4 bar pressure “Reprinted/adapted with permission from Ref. [65]. Copyright © 2020, Springer Nature”.

Conversion of non-printable material into a printable formulation is a challenging task in 3D printing technology. Nida et al. [65] explored the 3D printing of rice husk fractions for food packaging applications. In this study, rice husk fraction wastes from milling industries were utilized to develop novel food packaging. While rice husk is not a naturally printable material, it was improved with the addition of guar gum as a binding material for better printability. The results showed that the desired printability was achieved with the addition of 1% guar gum and printing conditions using a nozzle size of 0.82 mm diameter at 4 bar pressure with a printing speed of 2100 mm/min and extrusion motor speed of 300 rpm (Figure 8). Food packaging materials made from biodegradable waste can significantly reduce the use of non-biodegradable petroleum-based plastics. Furthermore, this research highlights novel and cutting-edge 3D printing applications for food packaging.

#### 5.2.2. 3D-Printed Food Packaging from Sugarcane Bagasse and Banana Peel

**Figure 9:**
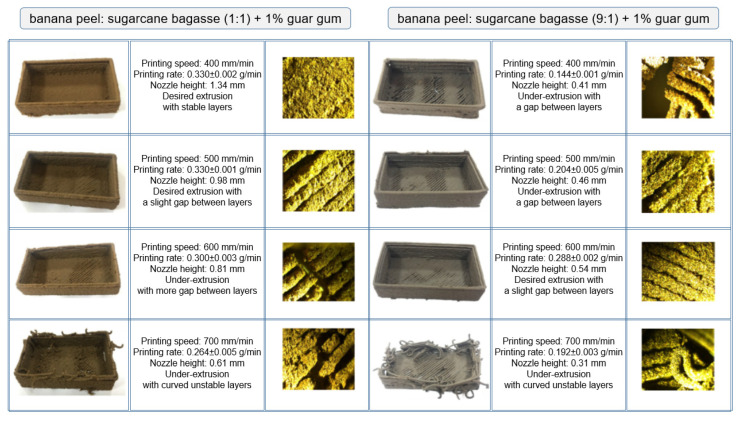
Optimization of material for 3D-printed food packaging from banana peel and sugarcane bagasse with 1% guar gum, using a nozzle diameter of 1.2 mm at 300 rpm motor speed and 3.2 bar pressure “Reprinted/adapted with permission from Ref. [66]. Copyright © 2022, exclusive license to Society for Sugar Research & Promotion, Springer Nature”.

Another case study on the application of 3D printing to food packaging for the valorization of agri-waste materials was reported by Nida et al. [66] using banana peel and sugarcane bagasse for food packaging development. Both raw materials are rich in cellulose, hemicellulose, and lignin, which provide a complex structure and stability for the 3D-printed forms. The study examined the effect of banana peel and sugarcane bagasse fraction printability at various ratios (1:1, 1:9, and 9:1) with the addition of guar gum for the customization of 3D food packaging. The results showed that the desired printability was achieved with the material supply at the ratios of 1:1 and 9:1 (banana peel:sugarcane bagasse) along with the addition of 1% guar gum and printing conditions using a nozzle size of 1.2 mm diameter at 3.2 bar pressure and extrusion motor speed of 300 rpm, with a printing speed of 400 and 600 mm/min for 1:1 and 9:1 (banana peel:sugarcane bagasse) combinations of material supply, respectively (Figure 9). In addition, Nida et al. [67] studied the applicability of sugarcane bagasse as an individual raw material and found the desired printability with a nozzle size of 1.28 mm and nozzle height of 0.45 mm, at 3.2 bar pressure with a printing speed of 500 mm/min, printing rate of 0.304  ±  0.003 g/min, and extrusion motor speed of 240 rpm. With the use of 3D printing, it is possible to customize materials based on the properties of the raw material, printing capacity, and packaging material, which differ significantly depending on the application.

#### 5.2.3. 3D-Printed Biomaterial Scaffold from Marine Bio-Wastes

**Figure 10:**
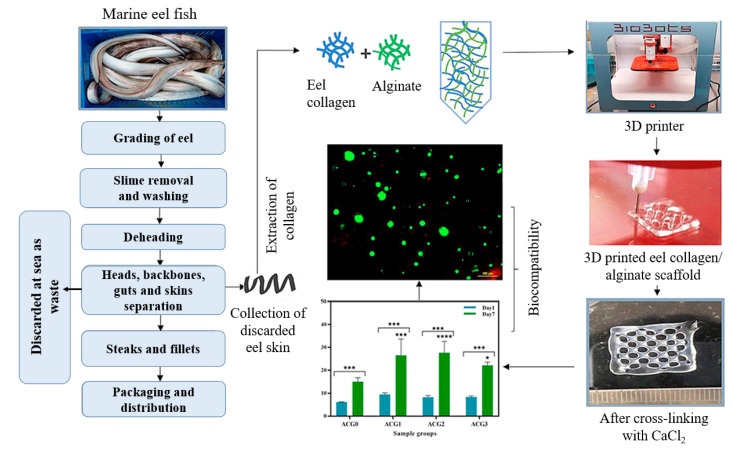
Biocompatible collagen-based scaffold from marine eel fish waste. * (*p* < 0.05) indicates statistical significance between different groups and different time points. “Reprinted/adapted with permission from Ref. [28]. Copyright © 2019, Elsevier”.

Fish-skin waste is disposed of in enormous quantities, which pollutes the environment. To address this problem, Govindharaj et al. [28] studied the development of a biocompatible collagen-based scaffold from discarded marine eel fish waste. In this study, the alginate hydrogel (5%) was formulated with different concentrations (0, 10, 20, and 30 mg/mL) of collagen obtained from discarded eel skin, and 100 mM calcium chloride was used as a chemical cross-linking agent. An alginate–collagen scaffold was printed in a layer-by-layer mode in a cuboidal shape (15 × 15 × 3 mm) using a nozzle size of 26 G at 20 KPa extrusion pressure (Figure 10). They also examined the biocompatibility of scaffolds and the feasibility of stem cell proliferation. The results revealed that the scaffolds exhibited excellent biocompatibility. Furthermore, the study reported that the 3D-printed scaffold showed improved metabolic activity based on the concentration of marine-derived collagen. A similar study conducted by Cestari et al. [68] reported the application of bio-hydroxyapatite synthesized from cuttlefish bones, mussel shells, and chicken eggshells for the development of 3D-printed poly(ε-caprolactone)/bio-hydroxyapatite scaffolds. In another study, gelatin and alginate extracted from lizardfish (*Saurida* spp.) scale waste and seaweed (*Phaeophyceae*), respectively, were utilized for the development of a 3D-bioprinted hydrogel scaffold [69]. The biocompatibility and nontoxicity of hydrogels derived from marine bio-wastes make them suitable for tissue-engineering applications.

#### 5.2.4. 3D-Printing of Food Waste-Reinforced Polymer Composite Filaments Cl_2_

**Figure 11:**
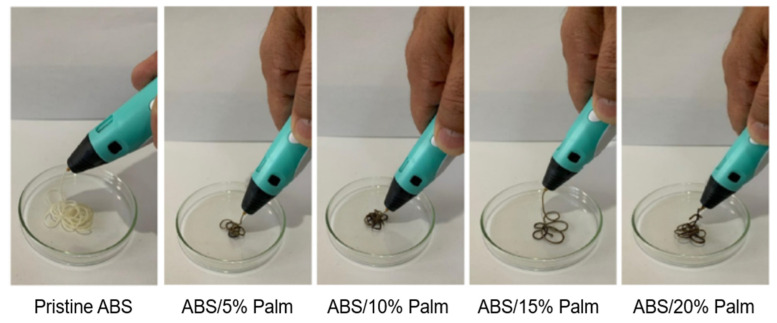
Fiber-reinforced filament composites from palm waste with acrylonitrile butadiene styrene using a 3D printing pen “Reprinted/adapted with permission from Ref. [70]. Copyright © 2022, Elsevier”.

Recently, many researchers have concentrated on using natural materials to find promising eco-friendly and economical materials. Reinforcing fibers from natural sources improve the mechanical properties of polymeric composites while also lowering their environmental impact. Marton et al. [70] utilized fiber-rich palm (*Archontophoenix alexandrae*) residues for the development of fiber-reinforced polymer filament composites. The study examined the effect on the printability of filament composites made up of acrylonitrile butadiene styrene (ABS) as a matrix with different concentrations (0, 5, 10, 15, and 20 wt%) of palm fiber using a 3D printing pen as a proof of concept (Figure 11). The results showed that the 3D-printed filaments had good appearance, shape, and stability. There was also no significant difference in printing behavior between pure ABS filament and fiber-reinforced ABS filament. Thus, the study emphasizes the promising novel application of natural fibers from leftover wastes for the development of mechanically stable and eco-friendly polymer filament composites using a 3D printing approach. A similar study reported by Lohar et al. [71] utilized walnut and eggshell powders for the development of waste biofiller-reinforced green hybrid polymer composite filaments made using polylactic acid (PLA) as a matrix material.

#### 5.2.5. 3D-Printed Green Composite Filaments from Fish Bone Waste

**Figure 12:**
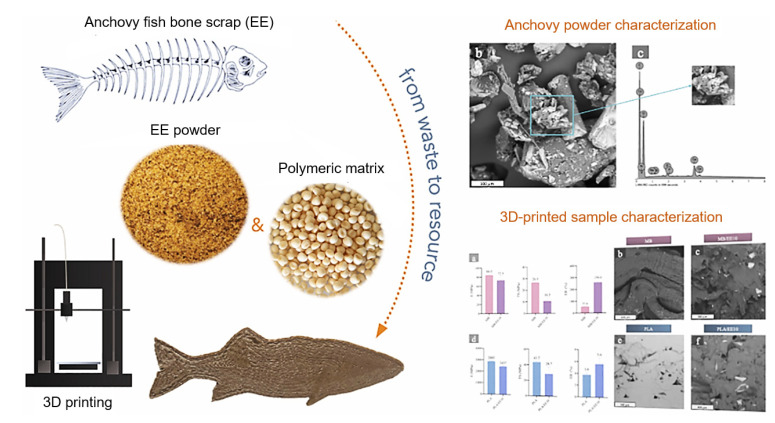
3D-printed green composite filaments from fishbone waste with polylactic acid “Reprinted/adapted with permission from Ref. [72]. Copyright © 2022, Elsevier”.

Fish bone is the major by-product of fish processing; it is considered a natural bio-composite made up of organic and inorganic materials that can be used to make eco-friendly plastics. Scaffaro et al. [72] studied the valorization of fish bone waste for the development of green composite filaments (Figure 12). They incorporated anchovy bone powder at different concentrations (10% and 20%) along with two different commercial polymers: PLA and Mater- Bi^®^ EF51L (MB). They studied the printability with a cylindrical nozzle to obtain a filament diameter of 1.75 mm, at nozzle and bed temperatures of 160 °C and 60 °C, respectively, with a varied infill rate of up to 100% and printing speed up to 50 mm/s. Furthermore, they characterized the morphological, rheological, and mechanical properties of the 3D-printed composite filament. The results showed the optimized infill rate and printing speed as 80% and 45 mm/s, respectively, for the desired extrusion of filament. Overall, a bio-composite filament with 10% anchovy bone powder showed excellent printability and stability.

## 6. Future Perspectives on Food Waste Valorization and 3D Printing

Waste valorization can contribute to the environment and society in many ways. A 3D printing approach to waste valorization, particularly food waste, can be promising. Firstly, the approach involves printing on demand, in turn reducing waste by producing more enticing and palatable edible or useful non-edible products. This is supported by the fact that 3D printing is an additive manufacturing process, explaining the great differences from conventional subtractive manufacturing processes in terms of resource utilization. In the context of the environment, 3D printing can offer other benefits in terms of scalability, tenability, energy usage, and safety. From a process economics point of view, it can be scalable, cost-effective, customizable in design, and a nonthermal approach. 

3D food printing must be both technologically and economically feasible, as well as appealing to consumers [73,74]. Agriculture and food processing industries generate a significant amount of edible waste. An idea that converts food waste into nutrient-dense foods can prove extremely beneficial. The advancement of technology in agri-food systems toward food security and nutrition necessitates a focus on utilizing and valorizing food waste for better applications. It is possible to make edible 3D-printed products out of food waste. However, consumers’ perceptions and preferences for foods from agri-food processing wastes also must be taken into account in exploring the creation of sustainable novel foods using 3D printing [5]. Consumers’ attitudes continue to be largely determined by their willingness to consume and their fear of new foods [73]. The benefits of 3D-printed foods can captivate consumers and can also have a big impact on their nutritional choices. The choices and preferences people have when it comes to food are incredibly varied and are influenced by a number of societal, cultural, environmental, and personal factors. In this context, perhaps, an attempt to taste novel 3D-printed foods made from unusual raw materials may be hampered by a few factors, including social and ethnic concerns, food neophobia, and the perception of 3D-printed foods as an unnatural method of processing regardless of the extent to which food can be presumed “real” or “food-like” [75]. Hence, to encourage people to accept this technology and these products, these attributes must be taken into account.

The market’s strategic vision, such as innovation economics and business planning of 3D printing applications, needs to be systematically monitored and controlled, which will help to predict the future societal effects of 3D printing. As a result, innovative thinking is required for policy implementation and decision-making. 3D printing is able to synchronously access digital design concepts at different production facilities due to the possibility of mass customization, which offers a lot of potential for increasing production volume [42]. Many business companies and industries are still investigating the technological possibilities of 3D printing [76]. Further research into the printability of food waste materials would be beneficial for developing new products. As a result, it is anticipated that 3D printing technology will promote significant improvement in economic growth in the coming years. The technological innovation of 3D printing supports a zero-waste economy through the collaboration of experts in waste management and the 3D printing industry [77].

With a focus on a circular economy, waste streams from one industry can be conveniently used as raw material for another industry in the development of innovative products. This provides a promising strategy to valorize wastes and by-products from agri-food processing. The food industry is influenced by rapidly developing digital technologies. The entry of 3D printing into the food sector provides enormous scope for the development of customized and personalized foods. As a manufacturing process, 3D printing is more integrated, allowing the effective use of energy and resources, in turn benefitting the environment by reducing waste production and carbon footprint. It is essential to note that detailed investigations of the techno-economic and socio-economic status of 3D printing are constrained. 

Ideally, the following questions must be addressed before venturing into the commercialization of this subject:

- Why take the 3D printing approach for the valorization of food waste?
- Are there any potential risks associated with the utilization of waste in edible products?
- What is required to promote the use of 3D printing to valorize food waste?
- Are food waste resources printable in their natural form? If not, how can they be converted into a printable form?
- Can fractions from food waste streams be utilized as direct resources or additive ingredients for 3D printing?
- How can 3D printing be used to create customized products from waste? Who is the end user?
- What about process times and costs? What about the investments to be made?
- Will raw materials be available year-round?
- What about the quality of products from food waste and the affordability of 3D printing technology?
- What is the possibility of upscaling 3D printing technology to reliable mass production?
- How versatile and sustainable is the technology?
- What challenges are associated with consumer acceptance of such foods?

## 7. Conclusions

Emerging 3D printing technology permits high levels of product customization and on-demand production. It also has potential advantages, including waste reduction and assistance with the value-addition of food waste. Concerns about agri-food waste, the revival of demand for food security, and growing interest in customized nutrition are all contributing to an increased awareness of sustainability. As an approach to food waste valorization, early studies indicate its promising potential. This article discussed the key highlights of such reports, explaining the scope for the development of customized foods as well as customized food packaging, among others. Food processing industry waste is a matter of global concern. Waste valorization approaches directly connect with sustainable food processing, especially considering the quantity and nature of such wastes. Among various valorization approaches, the idea of involving 3D printing technology is novel and requires significant research focus, in addition to addressing a sustainable approach to food waste management. To support future interventions, this article concludes with a series of questions to be answered before the commercialization of the technology is implemented. Overall, given the uniqueness and merits of the approach, in a broad context, 3D food printing has a strong possibility of transforming our food manufacturing processes. 

## Figures and Tables

**Table 1:** Selected examples of resources in food-processing waste streams, valorization approaches, and end uses.

Food Waste	Valorization Approach (Key Process Involved)	Product/High-Value Resources from Food Waste	Application	References
Red pepper waste	Spray drying and freeze drying	Phenolics and carotenoids	Health supplements and functional food additives	[16]
Carrot waste extract	Spray drying and freeze drying	Carotenoids	Bioactive ingredients/colorants in nutritional food formulations	[17]
Maize waste	Spray drying, freeze drying, and microwave drying	Phenolic compounds	Food and pharmaceutical applications	[18]
Beetroot pomace	Freeze drying	Bioactive compounds and pigments	Bioactive antioxidants for pharmaceutical applications and pigments/colorants for food applications	[19]
Passion fruit peel extract	Freeze drying	Polyphenol-rich powder	Functional food additives	[20]
Blackberry wastes	Fluidized bed drying	Blackberry granules	Dietary fiber and antioxidant-rich health foods	[21]
Grape pomace	Hot-air drying	Grape pomace powder	Formulation of functional muffins	[22]
Artichoke waste	High-pressure homogenization	Artichoke dietary Fiber	Dietary fiber-rich health foods or therapeutics against cadmium poisoning	[23]
Banana peel waste	Microwave-assisted extraction	Pectin	Thickening and stabilizing agents in food applications	[24]
Tomato waste	Enzyme-assisted extraction	Lycopene	Valuable ingredient for food and nutraceutical applications	[25]
Fish wastes and by-products	Ultrasound-assisted extraction	Fish protein and its derivatives (essential amino acids)	Functional ingredient for food fortification	[26]
Broccoli stalk	Acid extraction	Pectin	Thickening and stabilizing agents in food applications	[27]
Marine eel fish skin	Acid extraction	Collagen	Collagen for tissue engineering applications	[28]
Agri-waste	Enzyme hydrolysis	Mannooligosaccharides	Act as prebiotics, cancer-cell antagonists, and anti-allergic medications	[29]
Pineapple peels and crown leaves	Fermentation by *Aspergillus niger* I-1472	Vanillic acid and vanillin (aromatic compounds)	Flavoring agents in food applications	[30]
Multi-food waste (fish and agricultural wastes)	Fermentation by *Saccharomyces cerevisiae*	Single-cell protein	Protein supplements for animal feed applications	[31]
Marine processing wastes	Enzymatic hydrolysis and extraction	Bioactive peptides and proteins	Health supplements and bioactive therapeutics	[32]
Bean dregs (soybean residue)	Partial de-slagging	High-fiber tofu	Protein and fiber-rich functional food	[33]
Sago pith waste	Wet milling	Starch	Thickening, gelling, binding, and stabilizing agents in food applications	[34]
Deproteinized shrimp shell waste	Ultrafiltration process	Chitin	Thickening, stabilizing, and antibacterial agents	[35]

## Data Availability

Not applicable.
